# Effects of Temporal Heterogeneity of Water Supply and Spatial Heterogeneity of Soil Nutrients on the Growth and Intraspecific Competition of *Bolboschoenus yagara* Depend on Plant Density

**DOI:** 10.3389/fpls.2018.01987

**Published:** 2019-01-11

**Authors:** Hongwei Yu, Nan Shen, Dan Yu, Chunhua Liu

**Affiliations:** The National Field Station of Freshwater Ecosystem of Liangzi Lake, College of Life Sciences, Wuhan University, Wuhan, China

**Keywords:** resource heterogeneity, intraspecific competition, growth performance, clonal plant, population density

## Abstract

Clonal plants may face various types of resource heterogeneity in their natural habitats; as such, spatial or temporal resource heterogeneity can affect the growth of clonal plants. Clonal plants can concentrate their organs in a smaller area where resources are high would cause heterogeneity to increase competition between plants. Most studies on resource heterogeneity have investigated the response of plants under a single density or by manipulating a single resource. Few studies have tested the effects of the heterogeneous distribution of two covariable resources on plant growth and intraspecific competition. A greenhouse experiment was therefore conducted to study plant responses to the spatial and temporal heterogeneity of the soil and water supply under a variety of plant densities (one, two, four, or six plants per container). The perennial clonal herb *Bolboschoenus yagara* was grown under different combinations of water supply patterns, soil nutrient distribution types and plant densities while maintaining the total water and soil nutrient availability per container constant. Compared with that at a relatively high plant density, soil nutrient heterogeneity resulted in significantly less total plant biomass and less-modified morphological traits when the plant density is relative low. At the highest plant density, compared with the homogeneous soil treatments, the heterogeneous soil treatments significantly increased the total biomass and R/S ratio. Water supply patterns also clearly affected plant morphological traits at the highest plant density. Furthermore, soil heterogeneity significantly increased intraspecific competition intensity at low plant densities, but did not significantly affect intraspecific competition intensity at higher plant densities. Water heterogeneity had little impact on intraspecific competition. These results suggest that the growth performance and intraspecific competition of *B. yagara* are more strongly affected by soil nutrient distribution rather than by water supply patterns and that competition for soil nutrients may increase plant sensitivity to soil heterogeneity.

## Introduction

Resources (light, water, and nutrients) exhibit spatial and temporal heterogeneity which is ubiquitous within natural habitats (Jackson and Caldwell, 1993; Gross et al., 1995; Ryel et al., 1996; Farley and Fitter, 1999). Owing to the plasticity of their various plant traits, clonal plants can adapt to changing environments (Sultan, 1987; Hutchings and de Kroon, 1994). For example, plastic foraging, by organism searches or rootlets in areas where nutrient levels are higher than those in low-nutrient quality areas, can lead to more efficient use of heterogeneously distributed resources (Wijesinghe et al., 2001; Hutchings and John, 2004; de Kroon et al., 2005; Guo et al., 2011). In addition, as a physiological response to heterogeneity, organs of foraging plants (such as rhizomes, stolons, and corms) can take up nutrients at greater rates in nutrient-rich areas (Jackson and Caldwell, 1996; Hodge, 2004). The clonal plants can transport resources through connecting spacers among connected ramets in homogeneous or heterogeneous resources habitats (Alpert, 1999; He et al., 2011).

Besides resources heterogeneity, clonal plants are exposed to many other environmental stressors such as plant density (Fransen et al., 2001; Li et al., 2017). Plant density has a significant effect on the growth and reproduction performance of individual, population structure, and competitive relationship (Grace and Tilman, 1990; Stevens and Carson, 1999). The effect of intraspecific competition depends on the plant density (Antonovics and Levin, 1980). In addition, intraspecific competition may be affected by resource heterogeneity (Day et al., 2003b; Wang Y.J. et al., 2016). Several studies reported that the effects of resource heterogeneity on intraspecific competition led to changes in competitive intensity under heterogeneous distribution of a single resource (Day et al., 2003b; Hagiwara et al., 2010). For example, light heterogeneity significantly increased the intraspecific competition intensity of *Duchesnea indica* (Wang et al., 2012).

The relative nutrient concentrations in different soil areas (hereafter referred to as “soil nutrient heterogeneity”) can determine the extent to which plants concentrate more nutrient-absorbing organs in areas where nutrients are high; in addition, the efficient forage for nutrients in high-nutrient quality areas may lead to increased biomass, ramets and root production in heterogeneous environments compared to homogeneous environments that have the same amount of nutrient supply (Gersani et al., 1998; Fransen et al., 2001; Day et al., 2003b). Therefore, soil nutrient heterogeneity can influence interspecific and intraspecific competition (Day et al., 2003b; van der Waal et al., 2011; Mommer et al., 2012). Because plants prioritize the investment of relatively greater amounts of biomass in areas where nutrients are high in heterogeneous environments, competition between ramets and roots of neighboring plants may increase in intensity in smaller soil areas (Fransen et al., 2001; Day et al., 2003b). However, the results of other? experiments involving *Festuca ovina* (Day et al., 2003b), *Hydrocotyle vulgaris* (Dong et al., 2015) and *Alternanthera philoxeroides* (Zhou et al., 2012) have indicated that competition between plants is not influenced by soil nutrient heterogeneity or that this effect is temporary.

The temporal and spatial heterogeneity of water supplies (hereafter referred to as “heterogeneity of water supply”) clearly affects plant biomass allocation (Fay et al., 2003; Hagiwara et al., 2008), further altering community structure and composition (Maestre and Reynolds, 2007). For the same amount of water input, a stable water supply (hereafter referred to as “homogeneity of water supply”) can promote plant root systems to absorb water more efficiently and thus grow larger (Novoplansky and Goldberg, 2001; Hagiwara et al., 2012). In contrast, many plants exhibit negative biomass growth under conditions of heterogeneous water supply because those plants compensate for periodic water shortages by greater investment in roots, thus they have less to invest in other parts (Novoplansky and Goldberg, 2001; Fay et al., 2003; Hagiwara et al., 2010, 2012). In addition, spatial heterogeneity or temporal variation in water availability can alter intraspecific competition of *Perilla frutescens* (Hagiwara et al., 2010) and *Iris japonica* (Wang Y.J. et al., 2016).

Most studies on resource heterogeneity have investigated the response of only one species or the entire community by manipulating a single resource, e.g., nutrients, water, or light (Fransen et al., 2001; Day et al., 2003b; Fay et al., 2003; Moore and Franklin, 2012; Dong et al., 2015). Few studies have tested the heterogeneity of two resources affects intraspecific competition among clonal plants (Wang et al., 2012; Wang Y.J. et al., 2016), as the effects of resource heterogeneity on the relationships between plants may be altered by the supply patterns of other resources (Maestre and Reynolds, 2007).

Thus, we investigated the effects of heterogeneity in soil nutrients and water supply on the growth of both individual plants and the entire population under a variety of plant densities, as a single plant or a population at different densities usually experience both types of resource heterogeneity in their natural habitats. To test the responses of clonal plants to soil heterogeneity and water heterogeneity at different plant densities, we conducted a greenhouse experiment involving clonal plants of the rhizomatous species *B. yagara* (Ohwi).

Specifically, we addressed the following questions:

(a) Does the soil nutrient heterogeneity and heterogeneity of water supply affect the biomass accumulation in *B. yagara*?
(b) How do morphological traits of *B. yagara* respond to resource heterogeneity?
(c) Is the intensity of intraspecific competition of *B. yagara* affected by resource heterogeneity?

## Materials and Methods

### The Species

*Bolboschoenus yagara* (Ohwi) is a perennial clonal herb in the Cyperaceae family; this species develops underground rhizomes that terminate in a globose tuber (Board, 2010; Hroudová et al., 2014). Plants of this species occur in wet habitats such as swamps and wetlands and are distributed mainly in the northeastern, northwestern and southwestern regions of China (Board, 2010).

### Experimental Design

**FIGURE 1:**
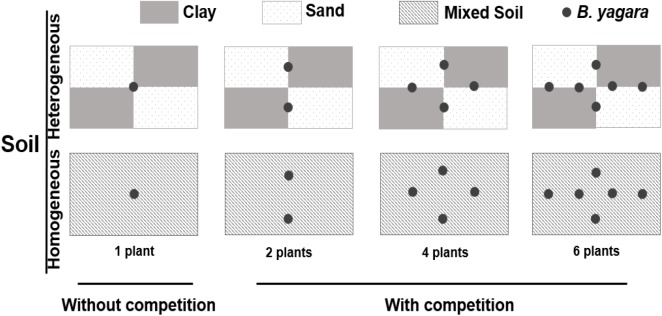
Experimental design. The experiment consisted of three factors. The first factor involved two patterns of water supply: a homogeneous (800 ml of water daily) and a heterogeneous (4 L of water every 5 days) water supply, with the total amount of water provided to each container kept constant throughout the experimental period. The second factor involved the intraspecific competition treatments: without competition (one plant per container) and with competition (two, four, or six plants per container). The third factor involved the substrate type. The first substrate represented the heterogeneous soil treatment, in which the containers were divided into four areas: two areas were filled with clay, and the other two were filled with true sand. The second substrate represented the homogeneous soil treatment, in which the containers were filled with the same completely homogeneous soil type. The total amount of soil nutrients was the same in all treatments.

On January 5, 2015, corms of *B. yagara* were obtained from mono-populations in a riparian area of Liangzi Lake, Hubei Province, China (30°05′–30°18′N, 114°21′–114°39′E). The corms were sprouted in sandy clay before the experiment setup. On April 1, 2015, 312 morphologically identical plants (without branches, height: approximately 12 cm; corm diameter: 0.91 ± 0.02 cm) were selected for the experiment described below, and 30 plants were randomly selected to measure their initial dry biomass (initial biomass: mean ±*SE*, 0.41 ± 0.02 g; corm biomass: 0.29 ± 0.02 g). The experiment involved a three-way factorial design. The first factor involved the pattern of water supply: homogeneous (800 ml of water daily) or heterogeneous (4 L of water every 5 days) water was supplied to each container, and the total amount of water provided was kept constant throughout the experimental period. Eight hundred milliliters equated to soil saturation, as measured by a soil moisture probe (SIN-TN8, Hangzhou, Liance Instrument, China). The environmental parameters of the water were as follows: total nitrogen (TN) concentration = 0.63 ± 0.009 mg.L^−1^; total phosphorus (TP) concentration = 0.04 ± 0.002 mg.L^−1^; pH = 8.55 ± 0.013; and salinity (SAL) = 0.09 ± 0.002 ppt [mean ±*SE*, measured by a YSI Professional Plus water quality meter (YSI Inc., Yellow Springs, OH, United States)]. The second factor involved the following four plant density treatments: one, two, four, or six plants per container. The third factor involved the substrate type. The first substrate represented the heterogeneous soil treatment. For this treatment, containers (70 cm long × 50 cm wide × 47 cm deep) were divided into four areas (35 cm long × 25 cm wide) (Figure 1): two areas were filled with clay (TN = 3.05 ± 0.05 mg.g^−1^; TP = 1.33 ± 0.03 mg.g^−1^; organic matter content = 60.67 ± 1.01 mg.g^−1^), and other two were completely filled with sand (TN = 0.02 ± 0.002 mg.g^−1^; TP = 0.25 ± 0.011 mg.g^−1^; organic matter content = 0.75 ± 0.02 mg.g^−1^) [mean ±*SE*, measured by a Flash 2000 Organic Elemental Analyzer (Thermo Fisher Scientific Inc., United States), IL500 TP Automatic Analyzer (Hach Corp., Loveland, CO, United States), and a Multiwave 3000 device (Anton Paar Corp., Austria)]. The second substrate represented the homogeneous soil treatment. For this treatment, containers were filled with the same soil type (comprised of equal volumes of clay and sand), after which the soils were completely homogenized. The total concentration of soil nutrients was the same in all treatments. Therefore, 16 treatment combinations (two water supply patterns × two soil nutrition distribution types × four plant densities) existed, and each combination was replicated 8 times. The mean temperature and mean humidity in the greenhouse were 25.34 ± 2.55°C and 64.67 ± 5.02% (mean ±*SE*), respectively. The experiment lasted for 70 days (duration of the pattern of water supply)—from April 2nd to June 10th 2015.

### Harvest and Measurements

The soil moisture (volumetric water content) and temperature were recorded with a soil moisture probe (SIN-TN8, Hangzhou, Liance Instrument, China) during the experimental period. The measurements were carried out daily before watering. To test for differences in the temporal heterogeneity of soil moisture between the two watering heterogeneity treatments, the soil moisture (*M*_m_) values, soil moisture minimum (*M*_min_) values and soil moisture maximum (*M*_max_) values were measured and recorded, and the temporal mean value of the relative soil moisture content was calculated (James et al., 2003; Hagiwara et al., 2010).

$$\mathrm{Relative soil moisture}=(M_{m}-M_{\min})/(M_{\max}-M_{\min})$$

We used one-way ANOVA to test the effects of the water heterogeneity treatment on both the temporal variability in the relative soil moisture content and the mean variance during the 5-day cycle in different water treatments. The mean value of the relative soil moisture was not affected by water temporal heterogeneity (*F* = 1.215, *P* = 0.275); however, the variance in the relative soil moisture during the 5-day cycle was significantly different between the water homogeneity treatments and the water heterogeneity treatments (*F* = 38.626, *P* < 0.001).

At harvest, plant height, fresh weight, rhizome length, ramet number, corm number and corm diameter were measured and recorded. The *B. yagara* material was subsequently divided into aboveground (leaves and stems above the soil surface) and underground parts (roots, corms and rhizomes). All the separated parts were oven-dried at 70°C for at least 3 days to obtain dry weights. To further calculate the performance of *B. yagara* at different densities, the root-to-shoot (R/S) ratios were calculated (Perez-Harguindeguy et al., 2013) as:

$$\frac{R}{S}\mathrm{ratio}(g\cdot g^{-1})=\frac{\mathrm{underground}\;(\mathrm{root mass}+\mathrm{corm mass}+\mathrm{rhizome mass})}{\mathrm{aboveground}\;(\mathrm{leaf mass}+\mathrm{stem mass})}$$

The effects of soil nutrient distribution and watering regimen on the intensity of intraspecific competition were calculated by the log response ratio (LnRR) of biomass (Armas et al., 2004).

$$\text{LnRR}=\text{ln}\left(\frac{\text{Bmono}}{\text{Bmix}}\right)$$

B_mono_ represents the total biomass in the absence of competition (i.e., solitary plant density treatment), and B_mix_ represents the average biomass of a plant per container in the presence of competition (i.e., multidensity treatment). The LnRR for each plant density treatment (2, 4, and 6) are calculated separately. The LnRR values are symmetrical around zero, and no ceiling is imposed on the maximum possible competition intensity (Goldberg et al., 1999; Weigelt and Jolliffe, 2003).

### Data Analysis

We measured biomass and morphological traits and calculated the R/S ratio and LnRR on a per-initial-plant basis for each container. All data was transformed by log_10_ prior to analysis to meet the requirements for homoscedasticity and normality. The treatment effects on plant height, corm number, corm diameter, rhizome length, ramet number, total mass, the R/S ratio and the LnRR were analyzed via a three-way ANOVA. One-way ANOVA in conjunction with Duncan’s (*P* < 0.05) test for *post hoc* comparisons was used to investigate the differences in biomass and morphological traits as well as in the R/S ratio, and the LnRR between the soil nutrient heterogeneity and the heterogeneity of water supply combinations at each plant density. To investigate the treatment effects on the intensity of competition, the LnRR was analyzed via a three-way ANOVA at each density. All of the analyses were conducted using SPSS 22.0 (SPSS, Chicago, IL, United States).

## Results

### Biomass and Biomass Allocation

**FIGURE 2:**
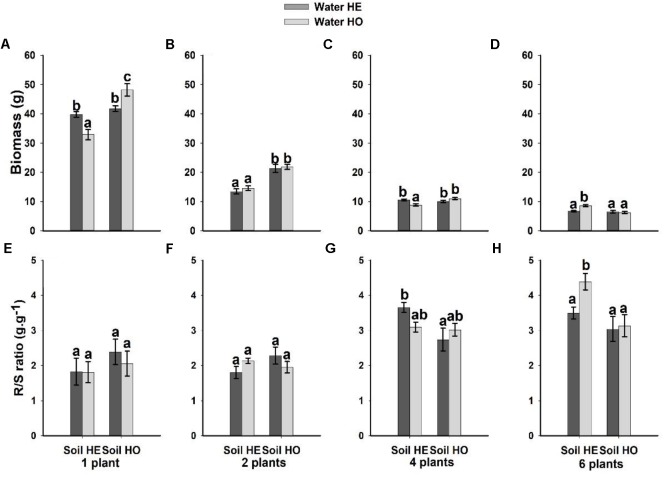
Effects of the heterogeneity of water supply and soil nutrients on the biomass **(A–D)** and R/S ratio **(E–H)** (±SE) of *Bolboschoenus yagara* at each plant density.

Both soil nutrient treatment (*P* = <0.001) and plant density (*P* = <0.001) significantly affected biomass, whereas water supply treatment (*P* = 0.351) did not (Table 1). The interactive effects between soil nutrient treatment and plant density (*P* = <0.001), and between water supply treatment, soil nutrient treatment and plant density significantly (*P* = <0.001) affected biomass (Table 1). The biomass was 37.5–55% larger under the homogeneous soil nutrient distribution than under the heterogeneous soil nutrient distribution in the one-, two- and four-plant density treatments, while the six-plant density treatment exhibited opposite results (Figure 2). The R/S ratio was significantly affected only by the density treatment (Table 1). The biomass allocation was not affected by soil nutrient heterogeneity at low plant densities (Figures 2E,F). However, the R/S ratios in the six-plant density treatments were greater under soil nutrient heterogeneity than under the soil nutrient homogeneity (Figures 2G,H). Also, there was no significant effect of water supply heterogeneity on plant biomass (Table 1).

**Table 1:** Three-way ANOVAs of the effects of water heterogeneity (W), soil heterogeneity (S), and plant density (D) and their interaction on biomass, the R/S ratio, corm number and corm diameter of *B. yagara*.

	Biomass (g)			R/S (g.g^−1^)			Corm number			Corm diameter (cm)		
	d.f.	*F*	*P*	d.f.	*F*	*P*	d.f.	*F*	*P*	d.f.	*F*	*P*
Water	1.80	0.881	0.351	1.80	0.105	0.747	1.80	0.551	0.460	1.80	5.334	**0.023**
Soil	1.80	36.000	**<0.001**	1.80	0.632	0.429	1.80	7.834	**0.006**	1.80	0.915	0.342
Density	3.80	1059.788	**<0.001**	3.80	32.325	**<0.001**	3.80	159.783	**<0.001**	3.80	196.861	**<0.001**
W × S	1.80	1.883	0.174	1.80	0.998	0.321	1.80	0.303	0.583	1.80	0.040	0.841
W × D	3.80	2.222	0.092	3.80	0.725	0.540	3.80	3.113	**0.031**	3.80	13.202	**<0.001**
D × S	3.80	29.590	**<0.001**	3.80	4.242	**0.008**	3.80	7.755	**<0.001**	3.80	6.071	**0.001**
W × S × D	3.80	9.462	**<0.001**	3.80	1.675	0.179	3.80	1.091	0.358	3.80	2.740	**0.049**

*Significant P-values are presented in bold.*

### Morphological Traits

**FIGURE 3:**
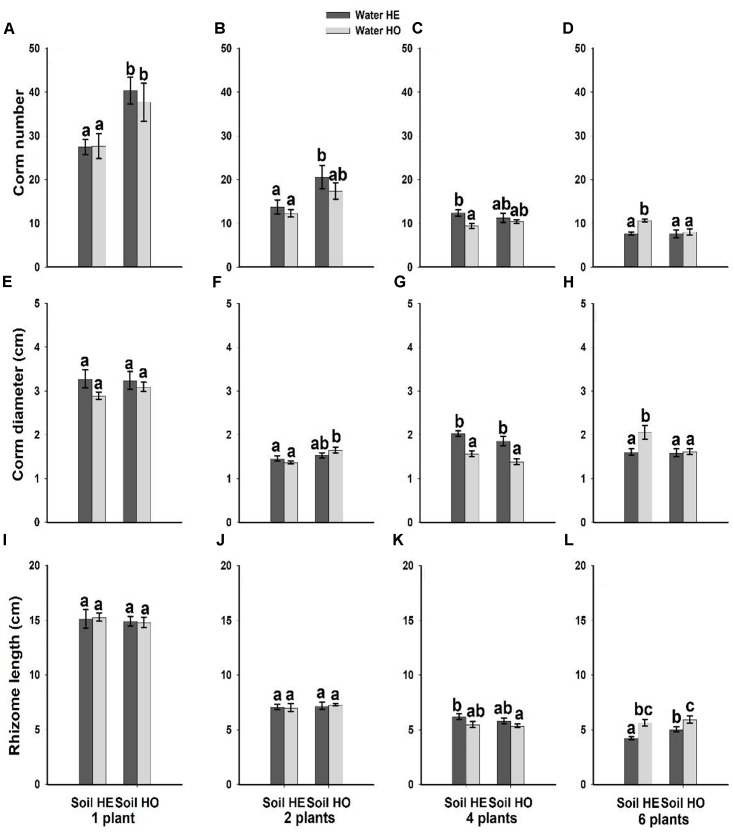
Effects of the heterogeneity of water supply and soil nutrients on the corm number **(A–D)**, corm diameter **(E–H)** and rhizome length **(I–L)** (±SE) of *B. yagara* at each plant density.

**FIGURE 4:**
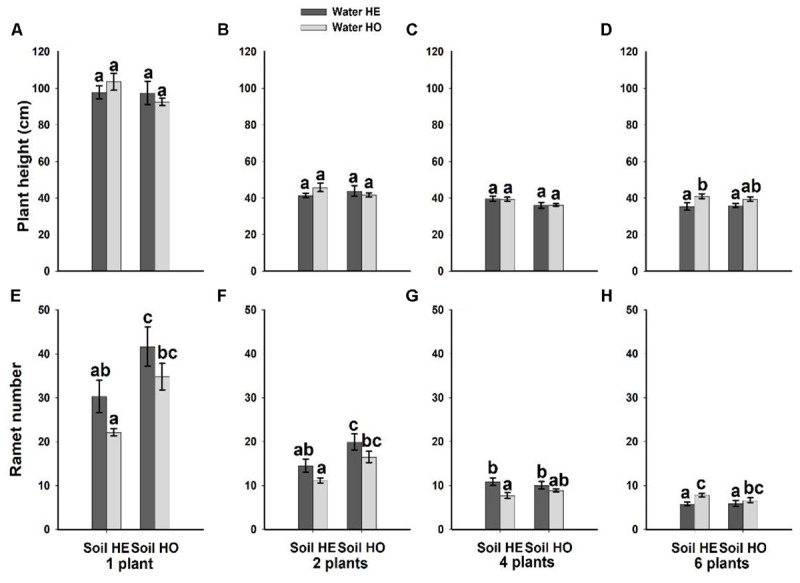
Effects of the heterogeneity of water supply and soil nutrients on the plant height **(A–D)** and ramet number **(E–H)** (±SE) of *B. yagara* at each plant density.

Compared with the water supply treatment the soil nutrient treatment significantly affected the morphological traits of *B. yagara* at low plant densities, although plant height and rhizome length were unaffected (Table 2 and Figures 3, 4). There were significant interactive effects between water supply treatment, soil nutrient treatment and plant density on corm diameter (Table 1). For example, compared with the soil nutrient heterogeneity treatment, the soil nutrient homogeneity treatment significantly increased the corm number, corm diameter and ramet number at low plant densities (Figures 3A,B,F, 4E,F). However, compared with the soil nutrient treatment, the water supply treatment significantly affected the morphological traits of *B. yagara* at high plant densities (Tables 1, 2). For example, with the exception of plant height, compared with the homogeneous water supply treatment, the heterogeneous water supply treatment significantly increased the corm number, corm diameter, rhizome length and ramet number of *B. yagara* at the four-plant density; however, compared with the heterogeneous water supply treatment, the homogeneous water supply treatment significantly increased the corm number, corm diameter, rhizome length, plant height, and ramet number of *B. yagara* at the six-plant density (Figures 3C,D,G,H,I, 4D,G,H).

**Table 2:** Three-way ANOVAs of the effects of water heterogeneity (W), soil heterogeneity (S), and plant density (D) and their interaction on rhizome length, plant height, ramet number, and LnRR of *B. yagara*.

	Rhizome length (cm)			Plant height (cm)			Ramet number			LnRR		
	d.f.	*F*	*P*	d.f.	*F*	*P*	d.f.	*F*	*P*	d.f.	*F*	*P*
Water	1.80	2.399	0.125	1.80	3.806	0.055	1.80	5.593	**0.020**	1.80	1.820	0.182
Soil	1.80	0.780	0.380	1.80	4.827	**0.031**	1.80	13.119	**0.001**	1.80	16.429	**<0.001**
Density	3.80	520.744	**<0.001**	3.80	509.556	**<0.001**	3.80	219.411	**<0.001**	3.80	346.382	**<0.001**
W × S	1.80	0.176	0.676	1.80	3.052	0.084	1.80	0.560	0.456	1.80	0.202	0.655
W × D	3.80	10.309	**<0.001**	3.80	1.922	0.133	3.80	5.566	**0.002**	3.80	2.335	0.106
D × S	3.80	2.419	0.072	3.80	0.828	0.482	3.80	6.142	**0.001**	3.80	38.437	**<0.001**
W × S × D	3.80	0.791	0.502	3.80	0.656	0.582	3.80	0.848	0.472	3.80	7.808	**0.001**

*Significant P-values are presented in bold.*

### Intensity of Competition

**FIGURE 5:**
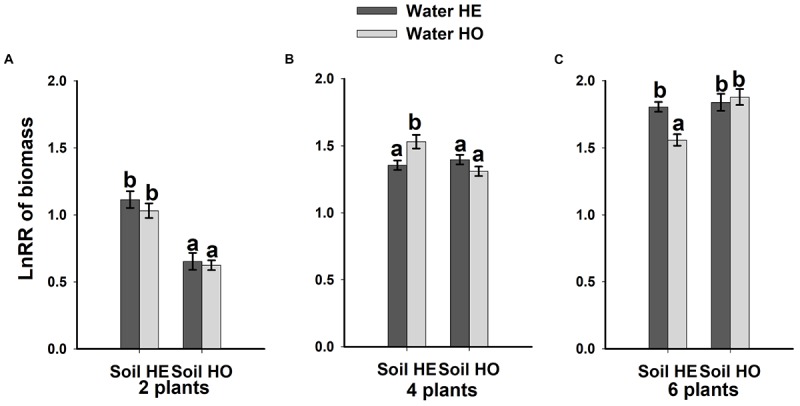
Competitive intensity as measured by the log response ratio (LnRR) of the biomass **(A–C)** (±SE) of *B. yagara* in response to heterogeneity of water supply and soil nutrients.

Compared with the water supply treatment, the soil nutrient treatment significantly affected the LnRR (Table 2). The interactive effects between water supply treatment, the soil nutrient treatment and plant density significantly affected the LnRR (Table 2). Compared with the soil nutrient homogeneity treatment, the soil nutrient heterogeneity treatment significantly increased the LnRR of the biomass at the two- and four-plant densities (Figures 5A,B). However, the opposite results occurred at the highest, six-plant density treatment (Figure 5C). These results mean that competition was more severe as plant density increased and was significantly and more strongly affected by the soil substrate heterogeneity than by the water supply heterogeneity.

## Discussion

### Biomass and Biomass Allocation

The results of several previous experiments have shown that some plant species accumulate greater biomass under heterogeneous conditions than under homogeneous conditions, given the same total concentration of available nutrients (Hutchings and Wijesinghe, 2008; García-Palacios et al., 2011). However, in the present experiment, plant biomass was greater in the homogeneous soil nutrient treatment than in the heterogeneous soil nutrient treatment (Figures 2A–H and Table 1). These patterns may result from the homogeneous conditions in which the nutrients are evenly distributed, which is therefore more conducive to plant growth (Maestre and Reynolds, 2007; Dong et al., 2012; Hagiwara et al., 2012), and partly due to physiological integration that allows ramets to share resources with other ramets (Dong et al., 2015). Plant density significantly affected the growth of *B. yagara*, as the average biomass of the plants gradually decreased as the density increased (Figures 2A–D and Table 1). These findings indicated that both the existence of intraspecific competition among plants and the competition intensity increased as the plant density increased, because of both the density-dependent effect attributable to increased numbers of competitors and the increased effect of competition on individuals in single-species plant populations (Antonovics and Levin, 1980; Watkinson, 1980).

Plants grow larger under a more homogeneous water supply than under a more heterogeneous water supply because they can take up water more consistently under homogeneous conditions that the low variability in resources availability, thereby allowing the plants to increase their growth performance continuously (Novoplansky and Goldberg, 2001; Hagiwara et al., 2010, 2012). In the present study, compared with the heterogeneous conditions, the homogeneous of water supply clearly led to more plant biomass when the plants grew in isolation (Figure 2A). In addition, we found that the heterogeneous of water supply did not affect *B. yagara* biomass accumulation, which may be because *B. yagara* is more sensitive to soil nutrients treatment than to water supply treatment.

Previous experiments have shown that some plant species employ morphological specialization and physiological responses to heterogeneity to place more nutrient-absorbing organs (e.g., roots or ramets) in nutrient-rich areas to forage efficiently for heterogeneously distributed nutrients (de Kroon et al., 2005; Gao et al., 2012). However, the R/S ratio of *B. yagara* increased only at higher plant densities under soil nutrient heterogeneity treatment (Figures 2G,H and Table 1). As the density increased, the plants were more likely to encounter resources deficits. Thus plants invested more in underground part rather than aboveground part to acquire resources (Day et al., 2003b; Hagiwara et al., 2010, 2012; van der Waal et al., 2011).

### Morphological Traits

Soil space decreased as the planting density increased, which caused the effect of soil nutrient heterogeneity to gradually diminish. Thus, *B. yagara* is more sensitive to water deficit at high plant density, and plants can alter their morphological characteristics according to the external environment. For example, plants have been shown to alter the length and angle of their spacers (includes stolon, rhizomes and corms, etc.), and their number and distribution of ramets (Hutchings et al., 2003; de Kroon et al., 2005; Gao et al., 2012).

Owing to high morphological plasticity, clonal plants generally respond positively to resource heterogeneity (Eilts et al., 2011; Zhou et al., 2012; Liu et al., 2017). For example, except at the 4-plant density, the *B. yagara* plants in the present study responded positively to resource homogeneity. That’s probably because the low variability in resource availability under the homogeneous conditions allowed the plants to absorb resources steadily, effectively improving their growth performance (Saeed and El-Nadi, 1998; Novoplansky and Goldberg, 2001; Maestre and Reynolds, 2007; Dong et al., 2012; Hagiwara et al., 2012). Overall, these results are consistent with other experiments that positive foraging responses to resource heterogeneity may not always be adaptive (Roiloa and Retuerto, 2006; Dong et al., 2015) and may be temporary (Day et al., 2003a,b).

### Intensity of Competition

Our results demonstrated that, compared with the heterogeneity of water supply, soil nutrient heterogeneity significantly affected the intraspecific competition of *B. yagara*. For example, soil nutrient heterogeneity increased the intraspecific competition at the two- and four-plant densities (Figures 5A,B). One explanation is that, to efficiently take up heterogeneously distributed resources, clonal plants place more nutrient-absorbing organs in nutrient-rich areas in heterogeneous environments. Also, the roots of neighboring plants would proliferate in nutrient-rich areas (de Kroon et al., 2005; Gao et al., 2012), thus competition becomes more severe under heterogeneous soil nutrient conditions than under homogeneous ones (Fransen et al., 2001; Day et al., 2003b). However, in the present study, heterogeneous soil nutrient conditions had no effect on the intensity of intraspecific competition at the six-plant density under a heterogeneous water supply. Other experiments have also shown that soil nutrient heterogeneity does not alter intraspecific competition at the container level for *Poa pratensis* (Maestre et al., 2006), *Achillea millefolium* (Rajaniemi, 2011), *A. philoxeroides* (Zhou et al., 2012) or *H. vulgaris* (Dong et al., 2015). These results may have been observed because resource heterogeneity can significantly affect plant competition when individuals are not genetically identical (Day et al., 2003b; Zhou et al., 2012). Other may be due to high resource depletion rate in the high density population, the nutrient-rich patches might gradually decline to the same level of suitability as the nutrient-poor patches, and then lead to high density population less sensitive response to soil nutrient heterogeneity (Roiloa and Retuerto, 2006; Dong et al., 2015). Thus, heterogeneity in soil nutrient availability has different effects on the intensity of intraspecific competition of *B. yagara* at different densities.

## Conclusion

We found that plants respond differently to environmental heterogeneity with respect to the supply of two covariable resources at different plant densities. The soil nutrient treatment significantly influenced the biomass and intraspecific competition of *B. yagara*. However, only the water supply treatment influenced the morphological traits of *B. yagara* at high plant densities, and heterogeneity of water supply had little impact on intraspecific competition. In addition, the interactive effect of soil nutrient heterogeneity and heterogeneity of water supply had no significant effect on the growth performance and competition relationship of *B. yagara*. Therefore, *B. yagara* was more sensitive to soil nutrient heterogeneity than to heterogeneity of water supply. Spatial or temporal heterogeneity in soil nutrient distribution and water supply patterns may be highly important with respect to the growth performance and population structure of clonal plants (Hutchings et al., 2003; Wang T. et al., 2016; Wang Y.J. et al., 2016; You et al., 2016). The ecological effects of resource heterogeneity should be investigated further due to various ecological factors (temperature, light, and humidity) that affect the growth performance of clonal plants. In addition, we should investigate how pulses of resource availability influence growth performance at individual, population, and community levels, because resource pulses provides opportunities to understand the dynamics of natural systems.

## Author Contributions

CL and DY designed the experiment and edited the manuscript text. HY and NS performed the experiment. HY and NS wrote the manuscript text and executed the technical assays and statistical analysis. All authors reviewed the manuscript.

## Conflict of Interest Statement

The authors declare that the research was conducted in the absence of any commercial or financial relationships that could be construed as a potential conflict of interest. The handling Editor is currently co-organizing a Research Topic with one of the authors CL and confirms the absence of any other collaboration.
